# Successful strategies for recruiting older African Americans into a dementia risk‐reduction trial The RAATE study

**DOI:** 10.1002/alz70858_107771

**Published:** 2025-12-26

**Authors:** Robert L Newton, Robbie Beyl, Floyd Hodoh, Miguel Hartford, Owen T. Carmichael

**Affiliations:** ^1^ Pennington Biomedical Research Center, Baton Rouge, LA, USA; ^2^ Aetna, Baton Rouge, LA, USA

## Abstract

**Background:**

African Americans are underrepresented in aging research. Few studies have assessed methods for recruiting older African Americans into clinical trials. The Reducing African Americans’ Alzheimer's Disease Risk Through Exercise (RAATE) study is a year‐long, randomized controlled trial that exclusively enrolled sedentary, cognitively healthy older African Americans and provides an opportunity to assess the effectiveness of various recruitment methods.

**Method:**

Community‐based strategies which included attending community events, churches, health fairs, and strategic placement of flyers were implemented by a designated recruiter. The academic institution implemented media‐based strategies including listserv emails, emails to past participants, social media, postcard mailouts, and advertisements on the institutional website, TV, and newspaper. Recruitment occurred from September 2019 – November 2024, with a year‐long pause due to the COVID pandemic.

**Result:**

There were 608 older African American adults who screened for the study and 129 were enrolled (mean age 68 +/‐ 5 years; 76% female; 34% with income < $50k/year; 45% married). The recruitment strategies that resulted in the greatest number of screeners included email (25.2%), friend/family (11.5%), community event (10.7%), past participant (10.2%), and social media (9.0%). The recruitment strategies that resulted in the greatest number of screeners included email (28.0%), family/friend (15.5%), social media (11.7%), past participant (7.8%), and community event (7.0%). Media‐based methods which are largely electronic, including email, social media, and contacting past participants, accounted for 44.3% and 47.4% of screeners and enrollees, respectively. Community based efforts, including community events, church, and health fairs were responsible for 27.5% of screeners and 16.4% of enrolled participants.

**Conclusion:**

The participant's high education level or the increased reach of electronic media into older populations may have contributed to the effectiveness of these methods. Community‐based efforts have historically been the most effective means of recruiting this population. It is believed that the pandemic limited the effectiveness of these efforts. These data suggest that a variety of strategies are important for recruiting an underrepresented group into a year‐long non‐pharmacological clinical trial.

